# Unveiling the potential of linseed mucilage, its health benefits, and applications in food packaging

**DOI:** 10.3389/fnut.2024.1334247

**Published:** 2024-02-07

**Authors:** Monika Chand, Rajni Chopra, Binanshu Talwar, Snigdha Homroy, Priyanka Kumari Singh, Aishwarya Dhiman, Abdul Wahid Payyunni

**Affiliations:** ^1^Department of Food Science and Technology, National Institute of Food Technology Entrepreneurship and Management, Kundli, Haryana, India; ^2^Department of Food and Nutrition and Food Technology, Institute of Home Economics, University of Delhi, New Delhi, India

**Keywords:** linseed mucilage, extraction, edible films, coatings, health, by-product

## Abstract

Industrial waste products derived from the oil industry often contain valuable substances and elements with great potential. These by-products can be used for various purposes, including as nutrients, bioactive compounds, fuels, and polymers. Linseed mucilage (LM) is one such example of a beneficial by-product obtained from linseed. It possesses favorable chemical and functional properties, depending on its method of extraction. Different pretreatments, such as enzymatic extraction, microwave-assisted extraction, pulse electric field, and ultrasound-assisted extraction, have been explored by various researchers to enhance both the yield and quality of mucilage. Furthermore, LM has exhibited therapeutic effects in the treatment of obesity, diabetes, constipation, hyperlipidemia, cancer, and other lifestyle diseases. Additionally, it demonstrates favorable functional characteristics that make it suitable to be used in bioplastic production. These properties preserve food quality, prolong shelf life, and confer antimicrobial activity. It also has the potential to be used as a packaging material, especially considering the increasing demand for sustainable and biodegradable alternatives to plastics because of their detrimental impact on environmental health. This review primarily focuses on different extraction techniques used for linseed mucilage, its mechanism of action in terms of health benefits, and potential applications in food packaging.

## 1 Introduction

Mucilage is a thick, gel-like material generated by plants, mainly made up of complex carbohydrates such as arabinoxylans, pectins, cellulose, and other polysaccharide variations (1). It plays different important roles in the plant’s functioning, such as retaining water, promoting seed germination, and providing protection against harsh environmental conditions (2). This slimy substance creates a shield around seeds when it comes in contact with water and turns into a viscous slime (3). It is predominantly found in the seeds, roots, and outer coverings of pods or leaves in a variety of plants, including linseeds, chia seeds, okra pods, psyllium husks, *Aloe vera* leaves, and more (4–6). Mucilage’s free hydroxyl groups form hydrogen bonds with water molecules, creating a thick, viscous matrix (7). This adhesive and thickening property makes it invaluable in the food and packaging industries. Mucilage’s versatility comes from its ability to bind, thicken, and retain moisture, making it an eco-friendly, nutritious, and flexible alternative to many existing food synthetic additives. In food, it serves as a natural emulsifier, stabilizer, and thickening agent, enhancing texture and shelf life in products like sauces and dressings (8). Its ability to form gel aids in encapsulating flavors and nutrients (9). In food packaging, mucilage’s adhesive nature provides a basis for biodegradable adhesives and the production of edible food coatings and films. It has shown protection against the permeability of oxygen and moisture and also has favorable functional attributes such as tensile strength and durability (4, 10–13). Its moisture-retention properties help prolong the freshness of perishable foods. Since packaging is utilized for every food product, including water, oils, spices, and baked goods, mucilage plays a significant role in the food sector. Keeping in mind the severe environmental pollution of plastic packaging, there has been a shift from the use of petroleum-based plastics toward biodegradable plant-based edible packaging (14).

Linseed (*Linum usitatissimum* L.) is also known as flaxseed, depending on its use as seed, oil (linseed), or fiber (flaxseed) (15, 16). It is one of the traditional crops grown and utilized since ancient times. It belongs to the genus *Linum* and the family *Linaceae* (15). It is cultivated in approximately 47 nations for seed, fiber, and oil (17). Worldwide, the Asian continent holds the largest share of 35.4% of the total production (18). The appearance of linseed is flat-shiny, and it possesses an oval shape. There are mainly two known linseeds: brown and yellow. Many products of the linseed plant are readily available in the food market in their various forms: whole linseeds, linseed oil, milled linseed, and roasted linseed (19–21). The linseed plant’s most valuable product is its oil, commonly known as flaxseed oil. Linseed is extensively used due to its health benefits, as it contains many nutrients and bioactives like fatty acids, minerals, vitamins, phenolic compounds, dietary fiber, and protein (22, 23). Linseed mucilage is one of the by-products of linseed. On average, linseed can produce mucilage ranging from approximately 3–10% of its total seed weight (23–26); however, the amount of mucilage produced can vary based on many factors, including the variety of seed and its cultivation process, environmental conditions during mucilage production, or the processing stage of the seed. The functional properties, such as good water-holding, emulsifying, fat-replacing, textural, stabilizing, and interfacial properties, of linseed mucilage make it an invaluable ingredient in various applications across the food industry, including gluten-free bakery products, plant-based meat and dairy alternatives, salad dressings, edible gels, and emulsions. Additionally, its biodegradability makes it an attractive choice for creating edible coatings and films that contribute to a more environmentally friendly food packaging solution without compromising on physical properties. These properties contribute to the overall quality and shelf life of food products while also meeting consumers’ demands for healthier and more sustainable options (27–30). LM not only has good physical functionality but also acts as a functional food. It exhibits a number of nutritional benefits, including laxative, anti-obesity, hypolipidemic, anti-diabetic, hyperglycemic, anti-cancerous, and other health benefits, including prebiotic, anti-bacterial, and anti-inflammatory effects (31–34). The underlying mechanism behind the immense health benefits of LM is still unexplored due to the limited availability of reported literature. Exploring and exploiting the potential of underutilized LM holds promise for revolutionizing both the food industry and sustainable packaging. This review sheds light on the possible applications of LM and highlights it as an ingredient, additive, or substitute for food products, as well as an environmentally friendly alternative for packaging materials. Understanding the nutritive value of LM is vital for advancing toward healthier food options and their application in nutraceuticals as the public becomes more conscious of their dietary choices in terms of their long-term impact on health. Furthermore, embracing LM as a solution for packaging materials can greatly reduce the environmental footprint of the food industry, promoting greener and more sustainable practices.

## 2 Methodology

**Figure 1:**
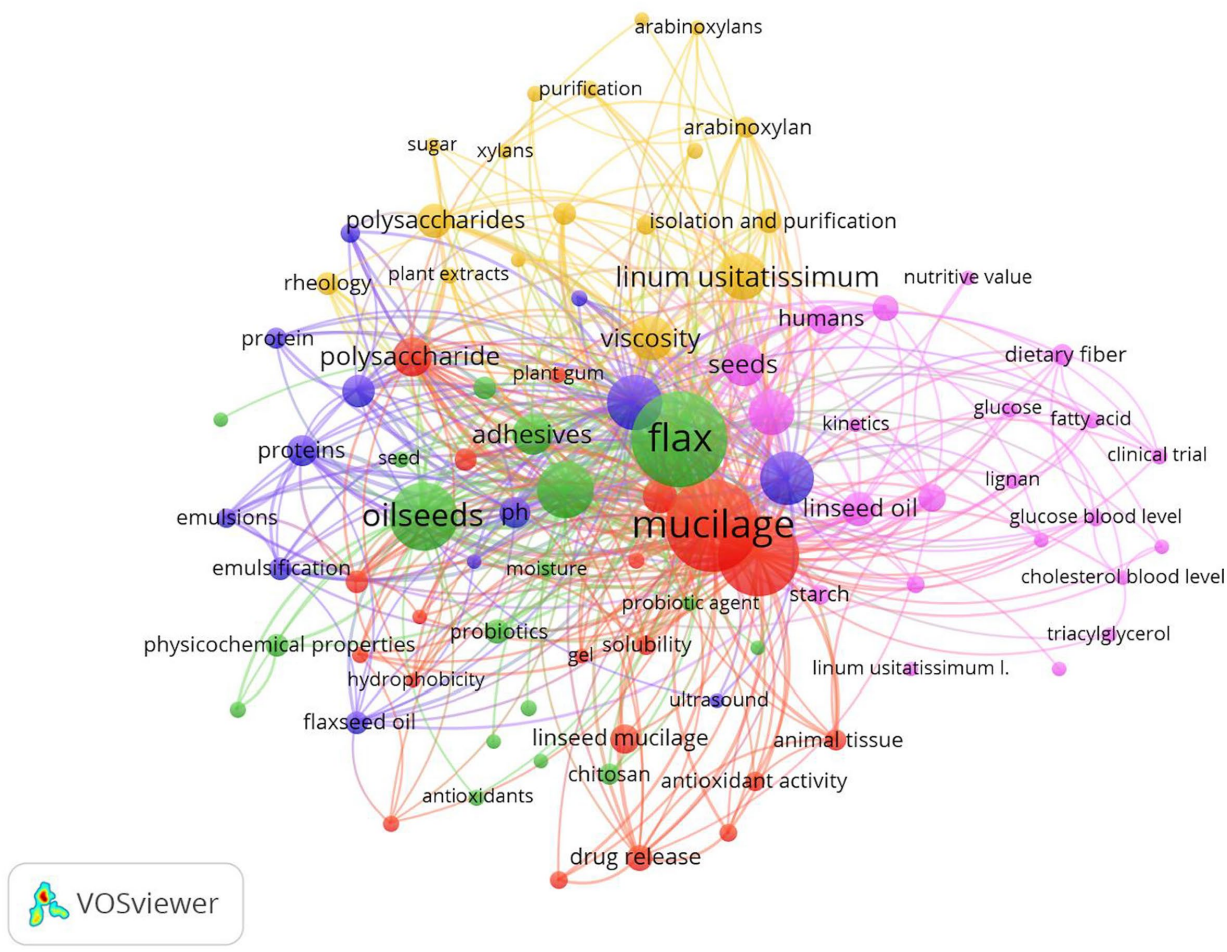
VOSviewer diagram with the analysis of co-occurrence of keywords indicating the publication trends on linseed mucilage (2000–December 2023).

The articles collected and reviewed were obtained through a search of articles, both review and research, indexed in the Scopus database from 2000 to 2023. A bibliometric analysis was conducted through VOS viewer software (version 1.6.19). The keywords given as a prompt were “flaxseed” and “linseed mucilage” in the software. The VOSviewer analysis of articles on flaxseed and linseed mucilage revealed five distinct clusters, highlighting current trends in research. The pink color represents the terms diet therapy, clinical trial, functional food, glucose blood level, and nutritional value, whereas the red cluster consists of mucilage, solubility, encapsulation, hydrophobicity, polysaccharide, and gel. The green cluster covers terms related to biopolymers, adhesives, antioxidants, tensile strength, and physiochemical properties. The blue color represents the terms chemistry, extraction, temperature, pH, and ultrasound. The yellow color represents the adhesive agent, rheology, viscosity, and molecular weight (Figure 1).

## 3 Nutritional and chemical composition

Linseed contains both soluble and insoluble fibers; its insoluble fraction contains lignin and cellulose, while the soluble fiber contains mucilage/gum, which is also well known as flaxseed gum (FG), linseed mucilage, or linseed gum (LG) (35). This soluble part of the seed is responsible for 6–10.2% of the mucilage content in linseed (21, 23). In comparison to other mucilage such as basil seed containing xylan (24.29%), glucan (2.31%), and glucomannan (43%), chia seed with glucose (19.6%), galactose (6.1%), arabinose (9.6%), xylose (38.5%), galacturonic acid (5.3%), and glucuronic acid (18.7%) (36, 37), and cress seed mucilage with glucose (1%), fructose (6.8%), arabinose (19.4%), rhamnose (1.9%), glucuronic acid (6.7%), and galactose (4.7%) (38). LM is composed of xylose (19–38%), galacturonic acid (21–36%), rhamnose (11–16%), arabinose (8–13%), galactose (12–16%), and glucose (4–6%). It is a heterogenic polysaccharide containing neutral and acidic parts, of which 75% is the neutral polymer, and has a molecular weight (MW) of approximately 1.2 × 10^6^ g/mol. The acidic part has two fractions of polysaccharides designated as Acidic Fraction 1 (3.75%) with a MW of 6.5 × 10^5^ g/mol and Acidic Fraction 2 (21.25%) with a MW of 1.7 × 10^4^ g/mol (39). The acidic part consists of l-rhamnose, l-fucose, l-galactose, and d-galacturonic acid in the ratio of 2.6, 1:1.4:1.7, while the neutral fraction consists of d-xylose, l-arabinose, and d-galactose in the ratio of 3.5, 6.2:1 (40). The rhamnose-to-xylose ratio, representing the acidic to neutral polysaccharides, might vary from 0.3 to 2.2, but the ratio is often around 0.7 (25, 40). This acidic-to-neutral polysaccharide ratio of linseed varies substantially according to its origin and source of extraction. The acidic polysaccharides have a smaller molecular size and exhibit Newtonian flow-like behavior, whereas the neutral polymer has a larger molecular size and shows shear-thinning flow (41). Furthermore, LM is also rich in minerals such as zinc (15.43–53.43 mg/kg) and copper (18.87–148.08 mg/kg). However, it has a lower content of chromium, lead, and cadmium (42) (Table 1).

**Table 1:** Proximate composition of linseed and LM.

Linseed					
S. No.	Fat (%)	Protein (%)	Ash (%)	Dietary fiber (%)	References
1	30–41	18–30	3–4	20–35	(43)
3	41	20	3.4	28	(44)
4	33.6	17.9	3.9	38.1	(45)

LM					
S. No.	Seed weight (%)	Protein (%)	Ash (%)	Carbohydrate (%)	References
1	5–5.3	20–24	10	30	(46)
2	3–9	4–20	3–9	50–80	(24)
3	3–9	4–20	3–9	50–80	(25)
4	5.56–6.54	7.68–12.33	–	24%	(31)

## 4 Extraction methods

**Figure 2:**
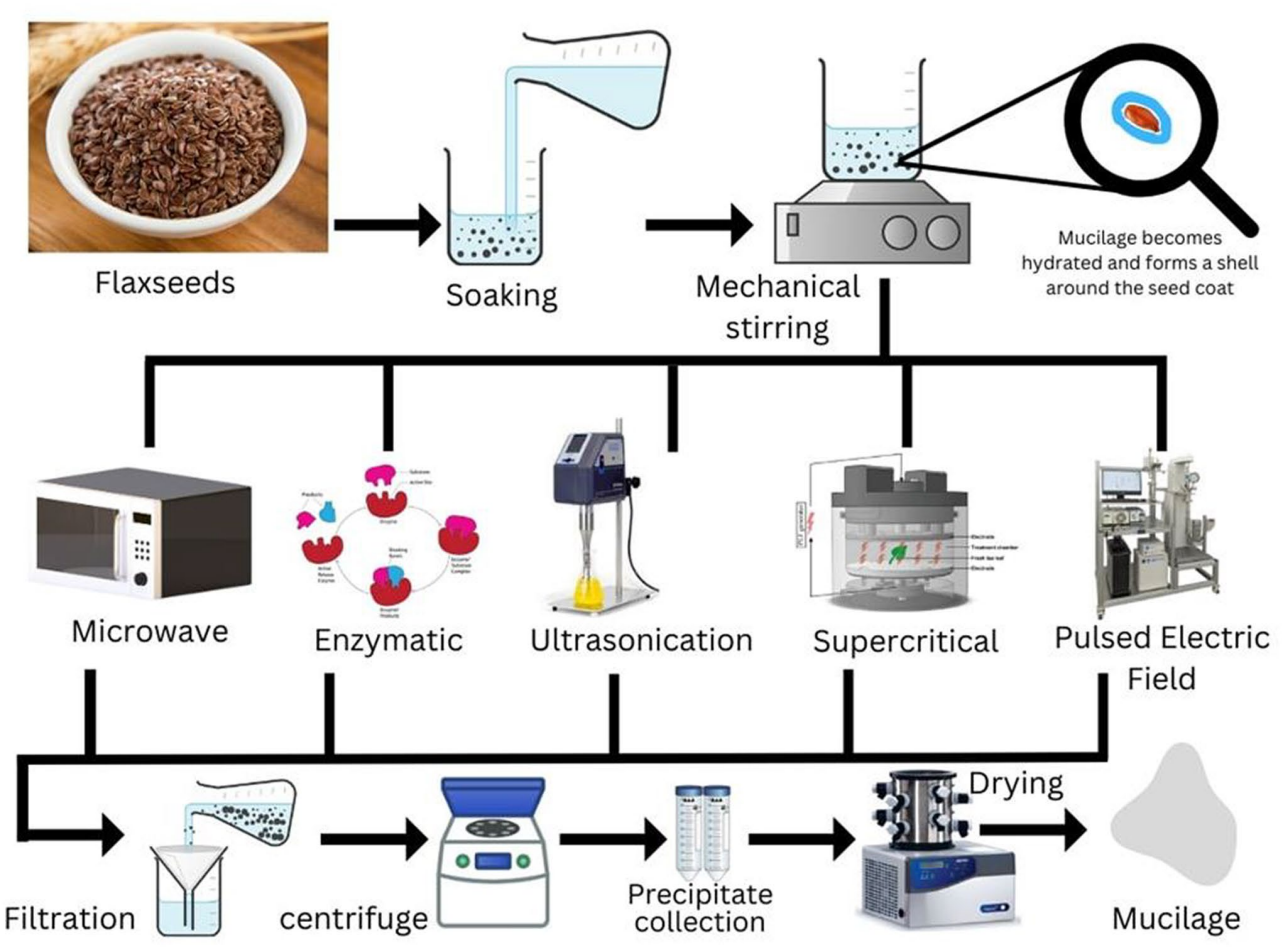
Schematic diagram for extraction of flaxseed mucilage and pretreatments.

Extraction of LM through aqueous extraction methods, such as hot extraction and solvent extraction, is affected by a number of factors, such as temperature, pH, mucilage content, seed-to-water ratio, ionic strength, and extraction method, which affect mucilage composition (47). The yield and purity of LM can be increased by different techniques, such as enzymatic extraction, microwave-assisted extraction (MAE), pulse electric field (PEF), and ultrasound-assisted extraction (UAE). Extraction process of LM is an important factor that substantially affects the final yield and functional, chemical, and rheological characteristics (48). Mucilage extraction involves two primary stages: maceration and precipitation. During maceration, the raw materials are immersed in a solvent or water at room temperature for a specific duration under controlled conditions. Both the duration and temperature of maceration have a positive impact on the proteins and molecular weight of the mucilage. Additionally, the use of solvents like acid, alkali, and EDTA has been found to enhance both the yield and quality of mucilage (49). However, it is worth noting that excessively high maceration temperatures and prolonged stirring can develop mucilage with an undesirable color, making it less suitable for commercial purposes. This can be prevented by acid pretreatments (5). Precipitation comprises drying the extracted mucilage for further use. Whereas, from a technological standpoint, extracting LM can be broken down into three crucial phases: raw material preparation, extraction, and recovery. Raw material preparation encompasses mechanical procedures such as removing impurities, husking, and screening. The extraction phase involves the release of mucilage, which occurs in two distinct stages: hydration and swelling. In hydration, the initial steps involve soaking seeds in the solvent/water. Once the linseed is hydrated, the mucilage undergoes rapid expansion, causing the external cell walls to rupture and form a thick mucilage capsule that separates the seed surface from its surroundings. This critical phase is influenced by agitation and thermal exposure, as these factors can affect the chemical bonds and facilitate the release of mucilage (2, 50). In the last recovery phase, the hydrated LM is separated through various methods, including filtration, scraping, alcoholic precipitation, or high-speed centrifugation (Figure 2). To improve the final yield, solvents such as isopropyl alcohol, ethanol, ethylene diamine tetra-acetic acid (EDTA) (49), sodium hydroxide (51), and hydrochloric acid have also been employed (7, 52).

A number of techniques have been used to extract mucilage, for which linseeds can be used as whole seed, crushed, or in dehulled form. Whole seed is a quick, effective water extraction procedure designed to enhance the removal of mucilage from whole seeds. This method is generally used before grinding and oil separation (39, 53–56). Crushed linseeds offer the main advantage of utilizing industrial by-products effectively, although they might reduce the quality of extracted mucilage due to the mixing of proteins. Earlier studies on crushed linseed included kefir fermented beverage yogurt and sourdough linseed polysaccharides (24, 39, 54, 55). Mucilage extraction from linseed hull is a more efficient method, but this method needs technical help as dehulling is extra laborious work and the transfer of oil during dehulling is a challenge (57, 58).

### 4.1 Aqueous extraction method

Aqueous extraction method, also known as wet extraction, involves well-established techniques used for generations due to its good yield and easy mechanism. Raw linseeds are soaked in water or a suitable solvent, followed by various mechanical and thermal treatments to facilitate mucilage release. Various solvents can be employed for extracting mucilage, including cold water, hot water, mild acidic, and alkali extraction methods. The acid and alkali extraction method has a higher mucilage yield compared to water extraction, attributed to the solubilization of insoluble polysaccharides within cell walls through de-esterification and subsequent elimination processes (25, 52, 59, 60). Aqueous extraction method, which considers the use of organic solvents, drastic temperature, and energy requirements during extraction, limits its application in the food industry; therefore, various pretreatments are employed as part of the extraction method, which gives high-value mucilage for edible use.

### 4.2 Role of pretreatments

These treatments are used for the extraction/recovery of functional compounds from the cell because of their unique mechanisms to disrupt cells, which include enzymatic extraction (EE), microwave-assisted extraction (MAE), pulse electric field (PEF), and ultrasound-assisted extraction (UAE). Among them, the highest yield was found in hot water extraction (HWE) (8.96%), followed by UAE (7.84%), MAE (7.01%), and at last, AAE (6.44%) (61). Whereas, the efficiency of UAE is better than magnetic stirring and microwave (52). The major challenges associated with these technologies are mainly running costs and good capital investment in their implementation and application.

#### 4.2.1 Enzymes

The underlying mechanism is based on the natural ability (specificity and regioselectivity) of enzymes to catalyze the hydrolysis of those components that are resistant to mass transfer, such as cell walls or binding to the target components, for example, pectin in the material matrix. When certain enzymes, such as cellulases, pectinases, and hemicellulases, are added to the extraction process, the structural integrity of the cell membranes and the wall is disrupted and degraded, enhancing the recovery of the target substances (62, 63). The enzymes used are specific to the cell wall composition. The effect of enzymes on cell wall breakdown and release of bioactive substances varied due to a number of factors, such as enzyme concentration and composition, solid-to-liquid ratio, type of solvent used during extraction, pH, enzyme/substrate ratio, time, and extraction temperature. For the extraction of LM, enzymes such as Pectinex Smash XXL, cellulase, β-glucosidase, and sulfatase are frequently utilized. The increase in extraction rate from 59 to 82% was accompanied by an increase in Pectinex Smash XXL from 50 to 200 μL kg^−1^ (64). This method has been widely applied in extracting bioactive compounds from linseed and yam (64–66).

#### 4.2.2 Microwave-assisted extraction

The microwave treatment works on the principles of dipole rotation and ionic polarization. The vibrational moments in dipoles and ions are responsible for their kinetic energy, which converts to heat energy due to frictional effects. The volumetric heating raises the extraction temperature and accelerates the mass transfer rates, thereby improving the extraction yield. The permissible operating frequencies of microwave systems are 2,450 MHz and 915 MHz, which are most frequently used for heating in industrial and residential settings (67, 68). Factors such as frequency, microwave power, irradiation period, particle size, moisture content, solid-to-liquid ratio, solvent composition and type, extraction pressure, extraction temperature, and number of extraction cycles affect MAE (69, 70). However, in contrast, other studies reported that linseed carbohydrates are not sufficiently agitated to allow for any noticeable improvement in their extraction, and a large amount of energy is wasted in heating the water molecules, making microwave-aided extraction the least efficient (51).

#### 4.2.3 Pulsed electric field

It works on the principle of electro-permeabilization, which forms pores and membrane breakdowns, leading to enhanced bioactive compound extraction (71–73). This technique enhances the solution’s solvent extraction and dehydration processes and increases the extraction rate by 50–80%. It was observed that there was an increase in yield up to 0.5 kV/cm, but higher power outputs ranging from 1 to 5 kV/cm did not result in a further increase in mucilage yield (74). Additionally, removing moisture from the material resulted in increased electrical conductivity, which may be due to the PEF-induced effect. Factors such as dehulling, milling, fractioning, the material’s electrical conductivity, the solvent used, frequency, pulse width, time, temperature, and wave shape affects PEF. The PEF requires more time to boost the effectiveness of the extraction, or a higher-energy PEF treatment may be necessary. However, this may have a negative impact on the structural integrity of bioactive materials since temperature increases throughout the treatment process are linked to bioactive materials (74).

#### 4.2.4 Ultrasonication alternative extraction

UAE is an alternative extraction technique with more benefits than traditional extraction and is usually exercised for the extraction of bioactive compounds, volatile compounds, polysaccharides, and essential oils from different sources, including spices, herbs, roots, and seeds (75–77). UAE aims to provide efficient energy consumption, better antioxidant properties, and reduced extraction time. Some researchers have adopted hurdle technology, like in grapefruit, to extract pectin but found that extended temperature and time might lead to these polysaccharides and protein degradation (78). Several process parameters affecting ultrasonification extractions include extraction time and cycle, the solvent’s nature, sample characteristics like matrix characteristics, particle size, solid-to-liquid ratio, chemical parameters acidity, pH, alkalinity, and temperature that varied the mucilage yield from 7.24 to 11.04% (79). UAE employed for LM extraction shows minimal impact on protein and monosaccharide composition and reduces LM’s intrinsic viscosity (61). The ultrasonic waves tend to improve the extraction efficiency, solubility, and foam stability of mucilage (80). Furthermore, this technique proved to be more effective than magnetic stirring and even microwaves, possibly due to its higher mass transfer coefficient and higher order kinetics (51) (Table 2).

**Table 2:** Role of pretreatment in mucilage extraction and its properties.

S. No.	Pretreatment	Study design	Major findings	References
1	Enzymatic extraction	Linseeds treated with Pectinex™, Ultra SP, Celluclast^®^ 1.5 L, and Viscozyme^®^ L	Decrease in content of polysaccharides and increase in protein recovery of treated linseed. Nitrogen solubility of defatted meals exposed with viscozyme increased.	(66)
2	Microwave	150 g linseed treated at 700 W for 1–5 min and various technological and functional aspects, changes in composition, and morphology of LM were studied.	Increase in mucilage extraction by +51%. Linseed exposed for 1–5 min microwaves resulted in augmenting but decreased the rheological, and functional properties if further extended the time	(81)
3	Ultrasound	Comparative analysis of UAE Magnetic stirring, and microwaves	UAE was the best among all the methods, as it decreases mucilage’s intrinsic viscosity and has a limited impact on polysaccharide and protein contents. Efficiency lies in this way UAE > magnetic stirring > microwave	(51)
4	Ultrasound	Hot water extraction [HWE], microwave-assisted extraction [MAE], ultrasound-assisted extraction [UAE], and alkaline–acidic extraction [AAE] were employed	Highest yield was found in HWE (8.96%) followed by UAE (7.84%), MAE (7.01%), and at last, AAE (6.44%). UAE maintains the purity of LM	(61)
5	Pulsed Electric Field	To study the effect of PEF on mass transfer kinetics of extraction of linseeds’ water-soluble polysaccharides Varying pulse numbers up to 900, p, fields strengths (0.5–5 kV/CM), and pulse duration 900 μs	Significant increase in mass transfer rate is found increasing by 50–80% in mucilage extraction	(73)

### 4.3 Factors affecting the extraction of linseed mucilage

Various technological parameters affect the yield of LM during extraction, such as pH, temperature, seed-to-water ratio, apparent viscosity, and protein content (82). With the increase in temperature, a reduction in water absorption and emulsifying capacity were observed (83). However, higher temperatures were associated with higher yield, ash, and protein content, which hampers the quality of the final mucilage (41). Therefore, moderate temperatures below 75°C were recommended to minimize protein denaturation (84). Additionally, pH significantly affects mucilage’s extraction yield and its fiber content. The maximum yield is obtained at the isoelectric point, and excessive use of acidic medium during the extraction of LM leads to the deterioration of mucilage. Acidic precipitation causes the protein residual matter in LM to decrease (e.g., acetic, trichloroacetic, and others). Although neither the sugar content nor the proximity of mucilage have changed during acidic precipitation, the LM extracted showed high thermal and mild acidic pH stability, lower surface charge density, and better solvation affinity, showing that LM is technically feasible for use in food product applications (84). However, the seed-to-water ratio is considered the other significant factor affecting mucilage yield (52). Seed-to-water ratio may lead to an increase or decrease in the viscosity of the medium and hence make it difficult to recover, which necessitates an optimal system dilution. Overall, temperature, pH, and seed-to-water ratio are the important factors that need to be considered for the extraction of mucilage (Table 3).

**Table 3:** Factors affecting mucilage extraction and its properties.

S. No.	Study design	Major findings	References
1	To optimize the three-stage countercurrent extraction process. The temperature ranges from 40 to 100°C for 0–60 min, and the mixer rotational speed is 0–240 rpm, followed by precipitation in three volumes of 95% ethanol.	This three-stage extraction for LM from whole seed at 80 ± 2°C, the seed-to-water ratio of 1:25, and the duration of each stage of 30 ± 1 min was formed to extract 98.3–99.1% of mucilage from linseed.	(52)
2	The mucilage ranges from 25 to 100°C for 0.5–8 h and seed-to-solvent ratio was 1:20 w/v Rotatory vacuum evaporator 40°C using 80% ethanol after 2–5 mL and then freeze-dried.	LM shows good foam stability in aqueous solutions.	(25)
3	To study properties of extracted LM (blinka variety) extracted at temperature ranging from 25 to 100°C using ethanol (1.4 v/v) evaporated at 40% (conc.) in a rotatory evaporator and freeze-dried	Extraction using water at 25°C yielded 3–5% mucilage while the mucilage yield was 8% when extracted using hot water. The best yield was obtained at 100°C for 8 h with seed-to-solvent ratio of 1:20 w/v.	(41)
4	The mucilage from different varieties was extracted at 85–90°C with a seed-to-solvent ratio of 1:13. After dialysis, the vacuum evaporator at 40°C was freeze-dried	Water-soluble polysaccharides fall in between 3.6 and 8%. Neutral monosaccharides fraction consisted of glucose, xylose, galactose, and rhamnose	(82)
5	To extract LM using the wet process at 70°C for 60 min and seed-to-solvent ratio was 7:1 and dried using vacuum dryer at 50°C	The wet process is time-consuming but can be scaled up as it is economical. Although some amount of loss in oil, protein, and SDG are loss in dehulling and demucilaging.	(85)
6	The sensory and rheological properties of LM extracted in bread. LM was extracted using hot water for 15 min and dried using a lyophilizer.	Rheology of LM-based dough was comparable to the starch, pectin, and guar gum Increase in sensory properties, limited effect on texture, and shelf life of bread.	(86)

## 5 Health benefits associated with linseed mucilage

**Figure 3:**
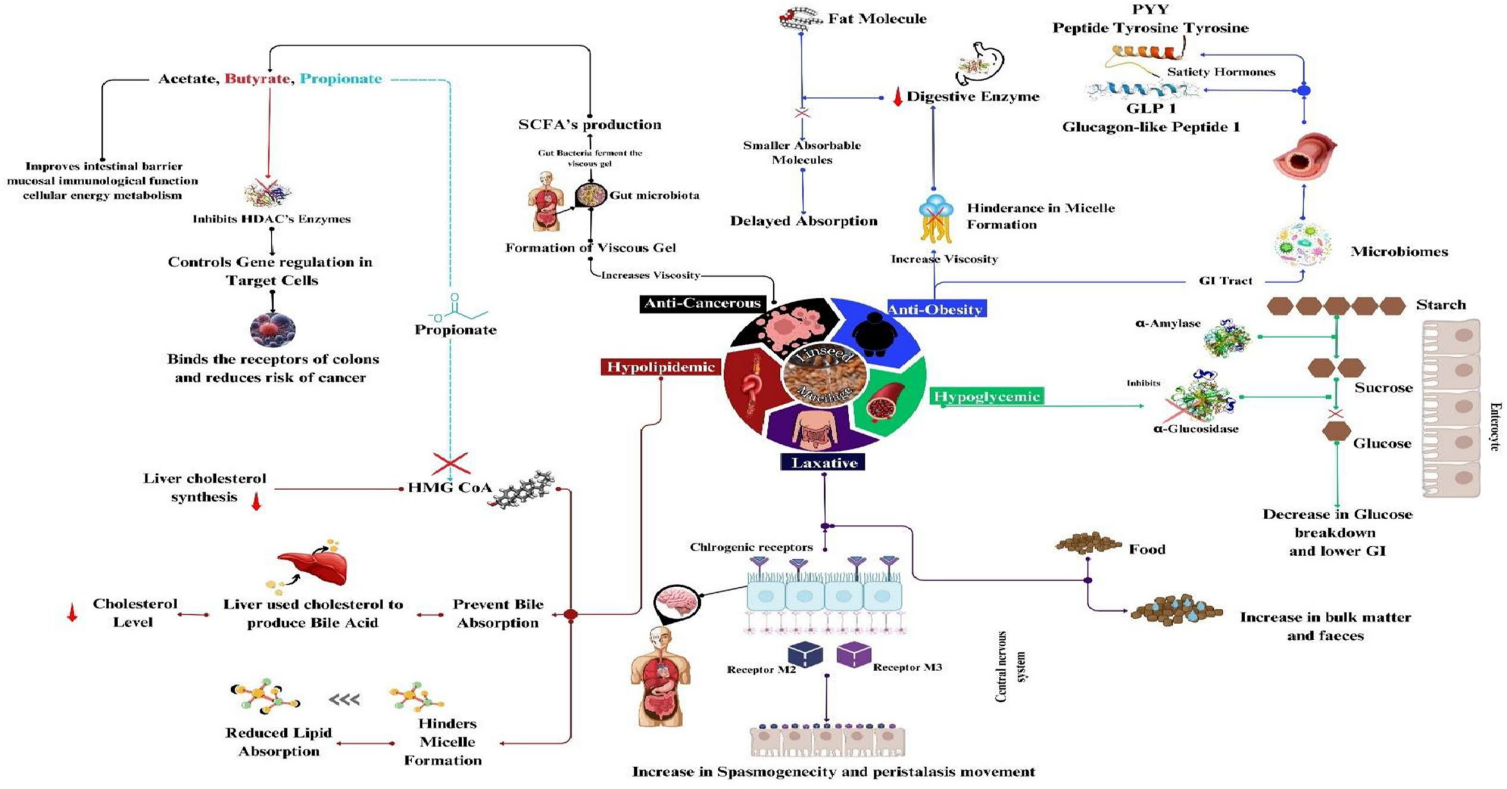
Mechanism action of linseed mucilage in the prevention of diseases.

LM is a therapeutic by-product that addresses a number of medical issues. Studies have shown remarkable health advantages of LM, including delaying gastric emptying, controlling glycemic load, anti-cancer effects, anti-ulcer effects, laxative effects, and reducing constipation (31–34). LM has a deep mechanism of action in the prevention of diseases through the modulation of various metabolites, as shown in Figure 3 (Table 4).

**Table 4:** Overview of health benefits of linseed mucilage.

Study design	Health benefits	Major findings	References
Carvacrol encapsulation produced from chia and Linseed mucilage against *Salmonella* and *Listeria monocytogenes*	Anti-microbial activity	Inactivation of *Salmonella* and *Listeria monocytogenes* by mucilage nanoparticles BIC of FMNP was 0.83 mg/mL against both microorganisms. Mucilage encapsulation could be a strategy to deliver carvacrol in foods.	(87)
To use LM to stimulate GIT condition Encapsulation of *Lactobacillus casei* in alginate microcapsules.	GIT conditions	The addition of LM enhances the survival of *L. casei* under gastrointestinal circumstances. LM affects positively the growth of *L. casei* in MRS broth, and 0.9% LM exhibited the highest viable bacterial count.	(88)
One sachet (LM) were served before or with main meals twice daily and for 12 weeks following a balanced but hypocaloric diet (20% reduction of individual’s daily energy requirements).	Anti-obesity	LM (IQP-LU-104) is effective and safe in body weight reduction at both dosages for overweight and moderately obese individuals. Significant weight reduction, waist and hip circumferences at the end of the 12-week study. 104 High dose group had a significantly higher reduction in body fat mass (4.25 ± 5.86 kg) than the placebo group.	(89)
To produce fat-free cream cheese fortified with probiotic bacteria and LM as a fat-replacing agent	Anti-bacterial activity	LM and probiotic bacteria worked against *Pseudomonas aeruginosa* and *Yersinia enterocolitica*. The product is fit for consumers with health issues related to fat consumption and sources of probiotic bacteria.	(90)
LM on the antioxidant, probiotic, and structural–mechanical characteristics of the different Lactobacillus cells.	Probiotic and antioxidant activity	LM elevates antioxidant activity, increases the resistance and survival of Lactobacillus cells in the GI tract, and leads to the synthesis of lipase and α-glucosidase inhibitors.	(91)
Effect of linseed mucilage on gastric lesions induced by ethanol in rats	Anti-ulcer	Significant reduction in the number and length of gastric ulcers induced by ethanol. The reduction in ulcer severity formed by an oral dose of linseed oil (5 mL/kg) was more effective than that obtained by ranitidine (50 mg/kg).	(31)
Dietary supplementation with LM in dogs: effects on apparent digestibility of fat and energy and fecal characteristics	Digestibility of fat/weight management	In dogs, LM decreased fat apparent digestibility and this effect was enhanced when combined with calcium. Dry matter and energy apparent digestibility were not affected	(92)
Linseed soluble dietary incorporated in prebiotic kefir	Prebiotic Antioxidant capacity	All kefir samples show the total bacteria counts were above 7.9 log cfu/ml at the end of the 28 cold storage period but LM-rich samples show 9.5 log cfu/ml. Enhanced lactic acid bacteria growth and antioxidant activity in kefir model.	(93)
The current study sought to determine intervention with *Lactobacillus paracasei*. Obese menopausal women were intervening with LM (10 g)	Insulin sensitivity	The intake of LM led to a reduction in serum C-peptide and insulin release during an oral glucose tolerance test and improved insulin sensitivity measured by Matsuda index	(94)
Effect LM was seen on constipation and diarrhea Oral administration of Flaxseed oil (30 and 70 mg/kg, orally) and mucilage (1 and 2.5 g/kg, orally)	Constipation and diarrhea	Feeding LM increases in wet feces in mice and shows laxative properties. Atropine completely blocked the effect of LM on isolated guinea pig ileum.	(31)
LM is a polysaccharide rich in anti-cancerous properties used in drug delivery material for cancer.	Anti-cancerous	The cytotoxicity of QUR@AA-g-FGS was investigated on HCT-15 cell line (cancerous human cell line) and, QUR@AA-g-FGS demonstrated outstanding toxicity toward HCT-15 cell line	(95)
Seventeen participants in a double-randomized crossover research were given three distinct isocaloric diets high in linseed fiber. The impact on fat, energy excretion, and appetite sensation were measured.	Appetite control	Significant effect on triacylglycerol, high mucilage meal fed showed smaller area curve compared to lower mucilage meal. Higher mean ratings of satiety and fullness were seen in high mucilage than in low mucilage.	(96)
Acute postprandial glycaemic response of puddings rich with mustard mucilage, fenugreek mucilage, and linseed mucilage were fed to at-risk patients for type 2 diabetes after overnight fasting.	Anti-diabetic	Significant decrease in the peak glucose and insulin levels as compared to the control pudding.	(97)
Rats for 5 weeks were fed on high, middle, and low doses of LM. The serum biochemical indices, body weights, fats, and metagenomic gut microbiota information were analyzed.	Anti-obesity	LM achieved appetite suppression by decreasing the relative amount of Firmicutes/Bacteroidetes ratio and also by regulating some bacteria.	(98)

### 5.1 Hypolipidemic

Cardiovascular conditions are a wide variety of diseases that affect the heart and blood vessels. One of its conditions is hyperlipidemia, which refers to a class of either inherited or acquired conditions characterized by high lipid levels in the human body. Globally, and especially in the Western hemisphere, elevated cholesterol is responsible for one-third of ischemic heart disease, resulting in an estimated 2.6 million deaths worldwide (99). Various studies have reported hypolipidemic properties of LM, and there could be a number of plausible mechanisms. LM hinders the formation of micelles and reduces lipid absorption, which may be related to the decline in hepatocyte production of VLDL (100). LM also prevents the reuptake of bile acids; more bile acids are produced in the liver, which diverts cholesterol from being used to make lipoproteins, lowering blood cholesterol. Another reason could be that LM speeds up bile excretion from the body and thus reduces LDL cholesterol (3, 101). LM surpasses through the small intestine’s digestive process and gets quickly fermented by the large intestine’s microbiota, leading to the production of short-chain fatty acids (SCFAs)—especially propionate, which inhibits the synthesis of cholesterol by reducing HMG CoA reductase activity (key enzyme synthesis of cholesterol) (102). Propionate can activate particular GPCRs, including GPR41 and GPR43, which are present in many types of organs, including adipose tissue and the liver. The regulation of lipid metabolism and cholesterol homeostasis have both been linked to the positive metabolic consequences of activating these receptors (103, 104). Linseed dietary fibers have been proven to be strongly fermentable in rats and humans, and results reported that the intervention of 5 g of dietary fibers from linseeds every day for a week significantly decreased total and LDL cholesterol by significantly increasing fecal fat extraction. Another possible mechanism of the hypolipidemic property of LM could be the effect of LM on bile acid metabolism (105). LM dissolves in water, resulting in the formation of viscous gels and, thus, increasing intraluminal viscosity. This modification of the gut lumen’s rheology could be one of the reasons that suggests the mechanical- or physical-based action of LM (104). Viscous fibers from *Aloe vera* leaf mucilage, psyllium husk, fruit, and vegetable fiber have also exhibited a hypolipidemic effect (106).

### 5.2 Hypoglycemic effect

Over the last several years, type 2 diabetes cases have soared, and the number of cases increased from 108 million in 1980 to 422 million, especially in low- and middle-income nations compared to high-income nations (107). There could be a number of factors, such as obesity, lack of physical activity, and smoking, responsible for diabetes. Since type 2 diabetes is characterized by hyperglycemia and impaired insulin sensitivity, carbohydrates have become a special dietary component of special importance.

Dietary fibers are well known to decrease the incidence of type 2 diabetes through glycemic management or reduced calorie consumption. LM is a polysaccharide compound that plays an important role in controlling diabetes. Studies have shown mucilage positively affects blood sugars in animals and humans (108). Soluble fibers delayed gastric emptying and decreased macronutrient absorption, resulting in lower insulin levels and postprandial blood glucose (109). Arabinoxylan (AX) is one of the soluble fibers found in LM; it quickly gets fermented by the colon’s bacteria in the GI tract. There is an inverse correlation between the amount of AX-rich bread consumed and the postprandial glucose response (103). The peculiar structure and rheological characteristics of LM, which have increased viscosity and improved swelling properties and help reduce blood glucose levels through a trapping mechanism, which is the main underlying mechanism for this impact, may be further explained by the inhibition of intestinal-glycosidases by mucilage (110). Also, this gelation process tends to retard enzyme mobilization for starch hydrolysis and glucose resorption (111). One such study, where adding 5 g of LM daily for 3 months lowered total and LDL cholesterol in type 2 diabetes by 10 and 16%, respectively (34) proves the importance of LM as an anti-diabetic agent. Different mucilage, like fenugreek seed mucilage and okra, have been attributed to the reduction in glucose levels (108, 112).

### 5.3 Anticancer

According to the WHO, cancer is the major cause of mortality worldwide, with more than 10 million deaths worldwide in 2020, and the most prevalent were found to be breast, lung, and colon cancer (113). Linseed is a rich source of both fibers (28%), such as soluble (1/3) and insoluble (2/3) fibers, whereas LM is a rich source of soluble fiber (114). Increased dietary fiber intake is linked to a lower likelihood of cancer. Soluble fibers in LM form viscous gel-like gooey substances in the colon that are easily fermented by gut microbiomes into short-chain fatty acids. LM can prevent cancer through several mechanisms, including bulking stool, speeding up transit time, and fermentation into SCFAs. Short-chain fatty acids enhance cell proliferation of the colonic mucosa, reducing the risk of colon cancer (104). Animal studies provide the link between SCFA and soluble fibers such as in mice and pigs, who were fed on a variety of linseed fibers and wheat. After incubation, gas production, and SCFA profiles were assessed, the amount of SCFA produced was highest in fecal samples obtained from animals fed with soluble LM compared to other fibers (115). Acetate, propionate, and butyrate are three SCFAs that are generated through the bacterial fermentation of soluble fiber in the intestinal lumen. SCFAs have several positive effects on the gut and general health, including acting as energy substrates for the gut. The most well-studied SCFA, butyrate, is responsible for many of the positive health benefits brought on by the intestinal fermentation of the mucilage. The SCFA butyrate improves its intestinal barrier, mucosal immunological function, and cellular energy metabolism and has some rather strong anti-inflammatory characteristics.

Butyrate can help prevent uncontrolled cell development and oxidative stress by inhibiting the enzyme histone deacetylase HDACs, which control particular genes (i.e., epigenetic regulation) in target cells (116, 117). BOHB (β-hydroxy butyrate) is an HDAC inhibitor; butyrate has comparable tumor-suppressing properties because it attaches to the same cell surface receptor in the colon. This reason could probably be one of the underlying processes through which the consumption of LM is thought to help lower the risk of colon cancer. In a supporting study compared to β-glucan control, linseed fiber treatment boosted SCFA synthesis and showed increased bacterial selection pressure (118). One clinical study proved that including 50 g LM/day for 4 weeks in adults could increase bowel movements per week by 30% compared to baseline (109). Other fibers, like psyllium husk, were also used for the same purpose. Both LM and psyllium products increase fecal bulk weight and are strongly linked with beneficial mechanisms that might improve colon health (119).

### 5.4 Anti-obesity

Over 1 billion individuals, including 340 million teenagers, 39 million children, and 650 million adults, are obese, which is still rising. According to the WHO, 167 million individuals, both children and adults, will be less healthy by 2025 due to being overweight or obese (120). This increases their risk of developing cardiovascular disease, diabetes, and cancer. One primary factor leading to obesity other than genetics and medical conditions is an increased energy intake-to-energy output ratio (121).

Dietary fiber can control calorie intake, facilitate weight loss, or maintain a healthy body weight (113). LM is a rich source of soluble dietary fiber and could serve as an anti-obesity agent. There could be a few proposed hidden mechanisms behind this. The viscous linseed fibers help lower body fat and control appetite satisfaction. LM is a soluble fiber that, on entering the large intestine, gets fermented and produces two hormones, glucagon-like peptide (GLP-1) and peptide YY (PYY); both of these gut hormones have a crucial role in inducing satiety (96, 122). Soluble fiber expands in the GI tract and transforms into a viscous substance that prolongs intestinal transit time, enabling thorough digestion and absorption. Food remains in the tract for longer, decreasing the next meal consumption (123).

Clinical trials provide evidence supporting that linseed mucilage, being abundant in soluble fiber, effectively contributes to the reduction of body weight (124, 125). Another possible mechanism responsible for this is the significant decrease in energy intake (126). The diets’ ME (metabolizable energy) content dropped as fiber intake rose. It should be noted that increasing fiber intake often lowers fat and protein digestibility (127). There could be a loss in weight with the consumption of LM, but this is accompanied by the type of diet (low or high fat) consumed (128, 129).

### 5.5 Other health benefits

#### 5.5.1 Laxative effect

Constipation is one of the gastrointestinal motility disorders associated with severe complications such as hemorrhoids, fecal impaction, rectal prolapse, perforation, anal fissures, and overflow diarrhea. These are some of the consequences of constipation. Constipation is a condition that affects 16% of individuals globally (130). Factors affecting constipation include type of diet, colonic motility, genetic predisposition, absorption, daily behaviors, socioeconomic status, pharmaceuticals, and biological factors, of which diet is the most important factor.

LM exhibits laxative properties, which are among its most well-known health advantages in treating constipation. The possible mechanism for this may be that an increase in the viscosity of the gastrointestinal content plays a vital role in defecation; due to its high water-binding capacity, it enhances the moisture level in feces, which causes a change in the quality, resulting in increased peristalsis (bowel movement). The other possible mechanism is via a cholinergic pathway that makes cell lines spasmogenic in nature (131). LM activates the chlorogenic receptors in the central nervous system (CNS) that stimulate the muscarinic receptors (M2 and M3) to produce spasmogenic action, enhancing peristaltic movement in the GIT (132). LM is a soluble fiber that helps delay stomach emptying and has a moderate laxative (133). Furthermore, LM helps reduce the porosity of ulcers as it is viscous in nature, which causes increased viscosity in GIT. This significantly decreases the length and number of gastric ulcers and acts as an anti-ulcerative agent (31).

#### 5.5.2 Prebiotics

Linseed mucilage acts as a prebiotic functional food, which might positively impact the human intestinal microbiota. This behavior facilitates changing bowel habits associated with the prevention of various illnesses like intestinal cancers due to its rich polysaccharide composition and high concentration of soluble heteropolysaccharides, which are the primary source of SCFAs. LM acts as a potential prebiotic and is known to confer health benefits to the host (8). However, it is noteworthy that the acidic fraction did not exhibit prebiotic activity, potentially due to the extensive branching of the xylose units. This branching structure may limit the accessibility of probiotic strains for fermentation. In contrast, the neutral polysaccharide fraction demonstrated significant potential for enhancing the growth of probiotic bacteria. Similar effects have been observed in mucilage derived from chia seeds (*Hyptis suaveolens* L.), psyllium seed, *Aloe vera*, basil, and *P. ovata* seed (134).

#### 5.5.3 Antioxidant effect

Antioxidants are the chemicals that eliminate/scavenge free radicals from the system (135). These have preventive effects on heart diseases, diabetes, cancer, and other severe complications. These antioxidants directly combat free radicals to prevent or minimize cell oxidative damage. Mucilage is a polysaccharide that exhibits natural antioxidant properties directly proportional to the mucilage dose (136). LM shows good antioxidant quantity as it is rich in phenolic compounds such as ellagic acid, cinnamic acid, caffeic acid, epicatechin, and vanillic acid (137–139). This behavior of LM positively impacts the gut as it helps synthesize α-glucosidase inhibitors and lipase, which in turn regulate the metabolism of the GIT tract (91). Other supporting studies associated with mucilage antioxidant activity were recorded in okra, quince seed mucilage, and *Opuntia ficus-indica* (140, 141).

#### 5.5.4 Anti-bacterial effect

Linseed mucilage may have anti-bacterial properties against many bacterial strains. This might be due to its specific bioactive compounds and complex composition. It is effective against *Escherichia coli*, followed by *Pseudomonas aeruginosa* and *Staphylococcus aureus* (142). Other mucilage, such as those obtained from chia seed mucilage-based films, act against *P. aeruginosa*, *E. coli*, and *S. aureus* (140). LM is a rich source of polysaccharides like arabinoxylan and rhamnogalacturonans, which might interact with bacterial cell walls and disturb membrane integrity. Moreover, the good water-holding capacity might trap bacteria and prevent adherence to the surface, decreasing the chances of colonization and growth. LM provides evidence and successfully protects *L. rhamnosus* GG from the harsh environment (87).

#### 5.5.5 Anti-inflammatory

Linseed mucilage has long been used as a nutritional supplement and in cosmetics. One of the reasons it is regarded as a useful component in the pharmaceutical and food sectors is due to its excellent anti-inflammatory action. A number of studies support that increasing dietary fiber, like LM intake, can decrease circulating levels of C-reactive protein (CRP), which is an inflammation marker in the body. CRP is also a predictor for CHD (coronary heart disease); the same relationship was established between dietary fiber and CRP (143). Furthermore, the DPPH scavenging activity and beta-carotene bleaching inhibition show LM to have strong antioxidant activity. These antioxidant properties of LM are thought to contribute to its healing or anti-inflammatory effect and are not dependent on the form of administration, whether it is applied topically or consumed orally (144).

## 6 Application in food

Due to its chemical structure and compositional features, LM acts as a gelling, thickening, binding, emulsifying, structuring, and fat-replacing agent (Table 5). Additionally, it shows good antioxidant potential and bioactive compounds, which makes it a valuable ingredient in functional foods and nutraceutical products. LM is considered a hydrocolloid with significant gelling properties; its addition improves the dynamic properties of the material, makes it stronger, and increases its ability to retain water due to increased hydrophobic interactions and cross-linking, which help in forming stable and consistent emulsions with a higher viscosity (25, 159). Mixing LM with other hydrocolloids increases the viscosity and has major benefits, such as reducing gel syneresis as observed in yogurt (154). Various factors, such as temperature, pH, and salt concentration, could affect gel strength. The dissolving temperature increased along with the gelling and melting points, and the addition of salt reduced the gel’s strength by lowering the Zeta potential. Various gluten replacers/structure-forming agents are used, such as hydrocolloids such as methylcellulose (MC), hydroxypropyl methylcellulose (HPMC), pectin, guar gum, xanthan, konjac gum, carrageenan, locust bean gum, agar, and psyllium gum, which are often thickeners and used as replacers in bread.

**Table 5:** Application of linseed mucilage in food products.

Product	Objective	Findings	References
Gluten-free bread	To study the influence of LM on the rheological characteristics of bread	Minor impact on texture characteristics and crumb The addition of LM at 1.8 and 2.4% conc. enhanced the sensory acceptance	(86)
Cookies	To replace butter in the low-calorie cookies with LM	Pseudoplastic behavior of LM With the increase in mucilage, there is an increase in Aw, antioxidant capacity, and overall acceptability (30%) Formation of 20% PM + 20% LM + 60% was considered best in prospect to high mucilage substitution, overall acceptance, antioxidant capacity, low fat, peroxide index, and firmness	(145)
Low-calorie cake	To replace LM with animal butter	With the incorporation of LM, tissue cohesion, antioxidant capacity, and resilience increased Decrease in elasticity, specific volume, and hardness of the shell. 60% LM + 28% flaxseed flour was an optimal sample with sensory, textural, and good nutritional value	(146)
Fat-free cream cheese	To incorporate LM as fat-replacing to formulate functional fat-free cream cheese	LM addition increases the protein, the total solids, and the ash Decrease in pH and moisture content Improvement in texture, overall acceptability, and enhancement of survival of probiotic bacteria.	(90)
Salad dressing	To formulate stabilizer in salad dressing	LM significantly improves viscosity and stability Salt contributed to the destabilization of the LM.	(147)
Elastic gels	To prepare hydrocolloid gels with flaxseed gum/konjac glucomannan and agar as a viscoelastic food.	As the FSG ratio dropped, the compound gel’s WSI value tended to rise. the food industry’s use of gelling agents based on FSG.	(148)
Cakes	To evaluate and produce low-fat cake containing LM and *Plantago Psyllium* mucilage as a fat replacer.	The best product was obtained at 30% LM together with 30% PM Lower volume, weight, specific volume, and height of cakes.	(145)
Ice cream	To use ethanol as precipitated LM and cress seed (CSM) and mucilage in ice cream production and compared with guar gum	Ice cream’s softening is more closely correlated with a rise in mix viscosity than an increase in overrun percentage. The optimum proportion to increase sensory and physical qualities was the addition of 0.025%	(149)
Meat and meat products	LM and its weakened gelling quality myofibrillar proteins	LM form stabilized and uniform emulsions, high apparent viscosity, strong disulfide bonds, and hydrophobic interaction. FG enhances the water-holding capacity, gel strength, and dynamic rheological properties. FG could be a potential approach to overcoming the deterioration of protein gels caused by catechin.	(149)
Peanut protein isolate (PPI) with flaxseed mucilage	The impact of LM addition on peanut protein isolate’s thermal gelation and rheological characteristics (PPI).	LM reduced the gelling time. FG-PPI gels the behavior of physical gels.	(150)
Stirred yogurt	To study the effect of LM and carboxymethylcellulose on the properties of stirred yogurt	Yogurt with added FSM + CMC had more viscosity and less syneresis. The yogurt that has been stirred has less cohesion and more adhesiveness due to FSM.	(151)
Mayonnaise	To incorporate LM, whey protein microparticles (WPMs) as a fats substitute	All samples showed shear thinning behavior and “weak gel” qualities. LM concentration sensory ratings for creaminess and mouth-coating increased, whereas those for hardness, fluidity, and spreadability dropped. Creaminess and mouth-coating sensory ratings rose with increasing FG concentration, but hardness, fluidity, and spreadability sensory scores dropped.	(148)
Coacervates	LM complex flaxseed protein isolate (FPI)-coacervates to encapsulate flaxseed oil (FO).	The best microencapsulation efficiency (95.4%) was seen in the microcapsules made using (FPI-HT)/FG complex coacervates. The microcapsules with the maximum oxidative stability were coated with (FPI-FPP)/FG complex coacervates.	(152)
Tea	Impact of LM on *L. rhamnosus* GG storage condition in hawthorn berry tea.	LM successfully protected *L. rhamnosus* GG from a rough environment.	(153)
Flaxseed mucilage cocoa milk.	The effect of LM, stevia, and US-formulated LM cocoa milk.	An increase in ultrasound treatment time decreases the viscosity. TPC and antioxidant activity of optimized FCM were notably higher and lower peroxide values.	(154)
Whey protein isolate	Whey protein isolate with linseed mucilage or almond gum	After heating, there was no change in droplet size, showing that the thermal stability of WPI had been significantly increased by conjugating it with SFAG and SLM.	(155)
Noodle	To formulate LM-rich noodles	LM improves the texture overall acceptability.	(155)
Whey fermented beverages	LM as thickening agents.	Beverage was found to have high antioxidant activity and free amino acids. LM enhances viscosity and bacteria survivability.	(54)
Biodegradable *Aloe vera* gel and Flaxseed mucilage	To study the effect of coating of plum fruits.	Fruit coated with *Aloe vera* gel with 0.05% LM powder yielded good results. Except for TSS and firmness, no other parameters were affected.	(156)
Emulsions: olive oil flaxseed mucilage	Characterization of olive oil and flaxseed gum emulsions. Olive oil emulsions (oil-in-water) 0.1–0.5% w/w	By adding 10% olive oil, LM made the emulsion more stable. LM-stabilized emulsions displayed superior rheological characteristics, creaming stability, and smaller droplet sizes.	(157)
Emulsion: soybean oil by flaxseed mucilage	Soybean oil stabilization by flaxseed mucilage and NMR characterization. Soybean oil emulsions 0.1–0.5% w/w	Emulsion particle size reduced as LM concentration increased; LM also showed gelling and thickening capabilities, and emulsions at 0.5% LM seemed to be a viscoelastic solid. Higher LM concentration emulsions had better structure and creaming stability.	(158)

**Figure 4:**
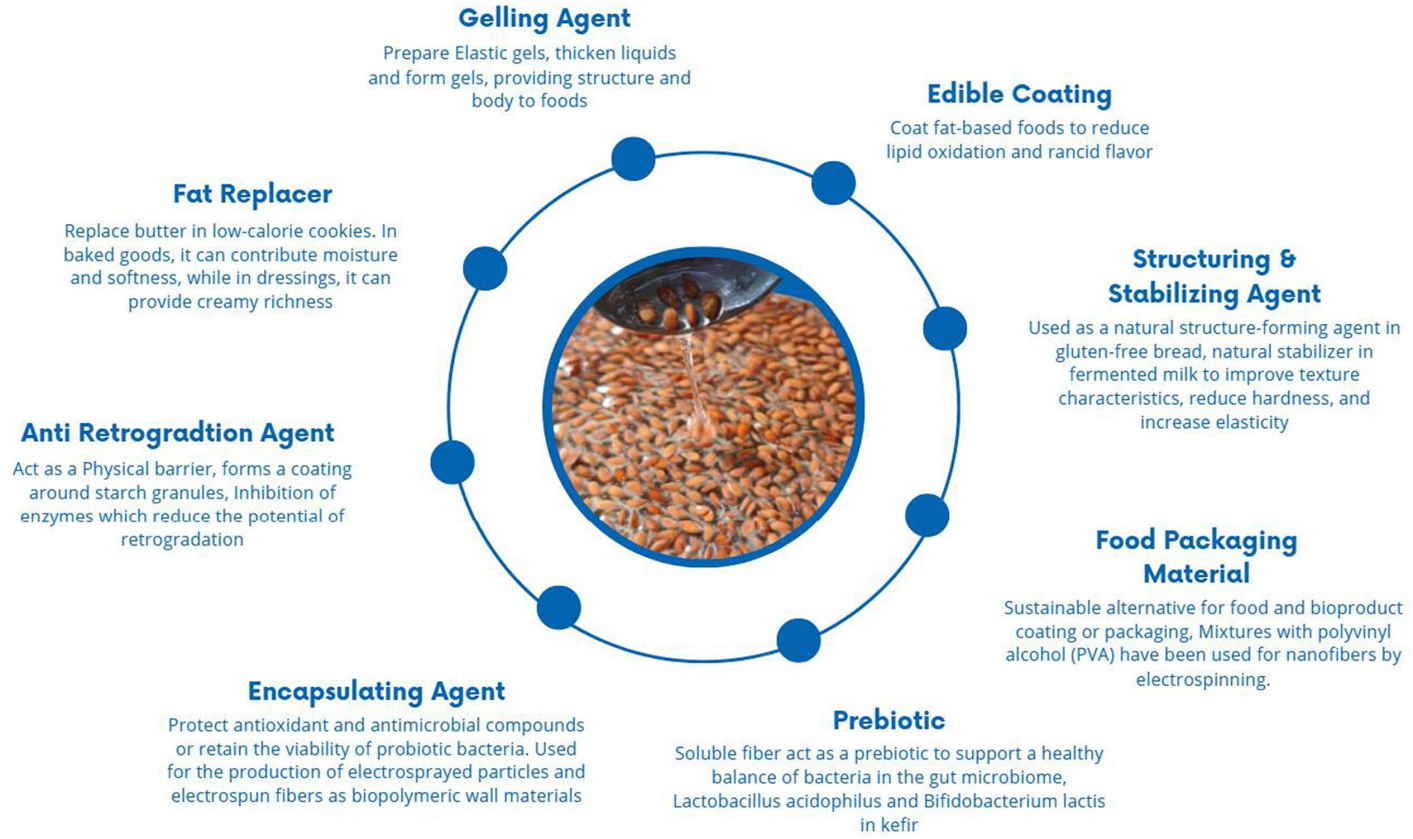
Application of LM in the food industry (86, 90, 139, 146, 151, 157).

LM incorporated in gluten-free bread enhances the bread quality of the dough as its arabinoxylan contents maintain gas cells during the initial baking stage, expanding the oven rise and enhancing the properties of the bread as its structure, loaf volume, crumb firmness, and texture (86, 160). Furthermore, incorporating LM exhibits comparable rheological characteristics to starch, pectin, and guar gum and contributes to enhancing the sensory attributes of bread (86). LM incorporated at high concentrations increases the viscosity of an aqueous dispersion and exerts a positive effect on surface-active proteins, but at higher concentrations, the increased viscosity can also reduce the free expansion of foam (161). Additionally, it enhances the meat product quality, and incorporating LM into starch can inhibit retrogradation by forming hydrogen bonds with starch molecules, which leads to increased water absorption, which helps to maintain the texture of starch gels and improve overall quality. LM is also used as a fat replacer to develop fat-free cheese; its incorporation reduces moisture content and increases viscosity (90). A number of mucilage-based products are commonly found in diets, including supplements, natural thickeners, pharmaceuticals, and personal care products (Figure 4).

## 7 Linseed mucilage in food packaging

Due to escalating environmental concerns and the global pollution crisis, the demand for biodegradable plant-based polymers (gums, mucilage, cellulose, and glucans) has garnered growing research attention. This shift is owed to their benefits, which include biodegradability, cost-effectiveness, and ease of production (162). The enhanced solubility, emulsion, hydrophobicity, barrier, and mechanical properties of polysaccharides, such as carboxymethyl cellulose, *Aloe vera*, chitosan, alginate, and pectin, successfully reduce the problem of quality loss of food products during storage (163–166). The food packaging industry is a huge sector, with its primary significance stemming from its ability to prevent the loss of nutrients and minimize microbial growth and other environmental contaminants, ultimately leading to product shelf-life extension. Edible packaging should possess certain mechanical and barrier qualities. An ideal edible package is generally colorless/tasteless, strong, and possesses low moisture and gas permeability. Some factors that make up the mechanical properties are tensile strength, elongation at break, deformability, and elastic modulus, as mentioned in Table 6 (177, 178). The mechanical properties depend on different environmental factors and the composition of the film. These properties are important to maintain the integrity of food products during processing and storage.

**Table 6:** Properties of linseed mucilage and other gums.

Mucilage type	Mucilage fraction	Composition	Concentration range (%)	Rheological properties	Intrinsic viscosity (dl g^−1^) and c [η]	Surface tension mN/m	References
*Asplenium australasicum*	Crude and PF-F	Glucuronic acid, neutral sugars (galactose, fucose, xylose mannose, and arabinose)	0.5	Not defined	18.86 and 29.11	55.9 and 62.2	(167)
*Descurainia sophia*	Crude	Mannose (49.91%), galactose (44.17%), arabinose (3.82%), and rhamnose (2.10%)	0.5–1.5	Psuedoplastic	7.82	Not defined	(168)
*Lepidium perfoliatum*	Crude	Callose, methyl-esterified homogalacturonan, and hemicellulose	1.5–3	Weak gel-like	Not detected	54–71 (CR) 58–71 (PF-F)	(169, 170)
*Lepidium sativum*	Crude	Arabinose, mannose, galacturonic acid, glucuronic acid, fructose, rhamnose, galactose, glucose	0.1–0.25	Pseudoplastic	3.92–1.45	054–71 CR 58–71 PF-F	(38)
*Linum usitatissimum*	Crude extract, neutral (N–F) fractions and, acidic (A–F)	d-galactose, d-xylose, l-arabinose, l-fucose, l-ramnose, d-galacturonic acid, l-galactose	0.1–2.5	Pseudoplastic for CR (>0.5%) and NF (>1%) viscoelastic	6.61 (CR), 6.43 (A–F), 6.68 (N–F)	55–72	(171, 172)
*Ocimum basilicum*	Crude, PF-F, high	Glucose, mannose, galactose, xylose, arabinose, rhamnose	0.1–0.3	Viscoelastic	39.2 and 0.22–0.91 (CR)	68–75 (CR), 72–79 (PF-F)	(173)
*Plantago major*	CR	Xylose, galactorunic acid, arabinose, glucuronic acid, galactose, rhamnose, and glucose	0.5–1	Pseudoplastic	14.08–1.22	63–69	(26, 174)
*Salvia hispanica*	Dry (D-CR) and wet (W-CR) extracted	Xylose, arabinose, glucose, glucuronic acid, galactose, galacturonic acid	0.25–1	Psuedoplastic and viscoelastic	Psuedoplastic	Not defined	(157, 175)
*Sinapis alba*	Crude	Galactose (13.8%), mannose (13.1%), rhamnose (4.5%), 6.9% uronic acid, and xylose (7.5%)	0.1–1.5	–	Not defined	47.5–65	(176)

CR, crude fraction; PF, pure fraction; AF, acidic fraction; NF, neutral fraction.

LM holds potential as an edible coating in food products (27, 28, 179). This application is based on its excellent functional properties, such as film-forming ability, moisture retention, biodegradability, water vapor permeability, adhesiveness, natural resistance to microbes, and various health benefits in the body (180). Moreover, LM shows excellent antioxidant, anti-bacterial, and antimicrobial properties that lead to enhanced packaging functionality. LM-based films not only increase shelf life from UV light during storage but also effectively maintain the viability of probiotics, reducing leakage and fruit softening and preserving sensorial properties (28, 31). The chemical reaction between the intermolecular bonds in the polymer chain (181), such as tertiary conjugate, was formulated using LM, gelatin, and oxidized tannic acid and showed the use of LM as wall material (9) (Table 7).

**Table 7:** Properties of packaging film developed using linseed mucilage.

Packaging film	Moisture retention (%)	Tensile strength MPa	Durability/elongation at break	Water vapor permeability	Opacity	References
LM with PVA (polyvinyl alcohol)	27–35	10.3–14.8	19.7–41.6%	1.5–3.04 (9.107) (g m^−1^ Pa h)	1.24–3.86 T_600_/mm	(28)
LM and PVA (polyvinyl alcohol)	At 70% relative humidity, it is 5.89–9.7 At 90% relative humidity, it ranges between 9.80 and 10.69.	7.18–17.30	1.62–4.14% Maximum force 37.60–73.60 N	1.62 × 10^−5^ and 1.90 × 10^−5^ g mm^−2^	Thickness 0.05 mm to 0.12 mm Opacity 6.49–9.54%	(182)
LM and glycerol	—	0.24–16.61 Film thickness from 0.05 to 0.22 mm	12.24–279.98% and Young’s modulus (0.08–174.77)	–	–	(183)
LM and PVA (polyvinyl alcohol)	27.61–35.28	10.3–19.7 MPa Thickness μm 35.5–47.0	19.7–416.2%	1.49–3.04 g m^−1^ Pa h	1.24–3.86 T_600_/mm	(184)

However, there are certain constraints because the films produced from pure mucilage lack the requisite mechanical properties to meet the food packaging requirements. Additional challenges associated with the use of LM in food packaging include high water solubility, high viscosity, and high swelling capacity. An effective way to ameliorate the physicochemical characteristics of LM coating is through blending modification (182). Numerous research endeavors focused on exploring the capacity of LM as an application in bioplastics, film formation, and edible coatings, as shown in Table 8.

**Table 8:** Exploring the potential of LM in food packaging.

S. No.	Study design	Major findings	References
1	Linseed mucilage LM + sodium alginate-based edible coating with bacteria *Lactobacillus casei* LC-01	Decrease in viability of the bacteria by an average, of 2.96 log CFU·g^−1^ under gastrointestinal situations. The LM base coatings enhanced the preservation and physicochemical characteristics of the vegetable while reducing darkening was found.	(185)
2	LM + chitosan (CH) combinations (LMCH) were applied on fresh-cut cantaloupe	Significant decrease in leakage and softening of juice reduced the microbicidal effect of CH Preserved overall sensory qualities and color and enhanced product acceptance up to 12–15 days.	(28)
3	Layer-by-layer edible coatings based on chitosan + pullulan (PU), Chitosan + LM, Chitosan + (NM) nopal mucilage, Chitosan + (AM) aloe mucilage was applied to fresh-cut pineapple.	Enhanced quality and extended the shelf life by 6 days. Whereas, decreased softening of pineapple, and weight loss, decrease in color and TSS content.	(186)
4	LM + lemongrass essential oil (LGEO) RTE pomegranate arils were produced	Application of coatings maintained the desired microbiological quality of pomegranate arils. With the increase in LM concentration of more than 0.6%, the coating solution became viscous. However, the combinations LM (0.6%) + LMEO (800 ppm) and LM (0.6%) + LMEO (500 ppm) were best in terms of sensory overall quality attributes.	(29)
5	LM (0.75, 1.0 and 1.25%) edible coatings Cheddar cheese during ripening at 8°C for 3 months	No significant impact on the growth of lactic acid bacteria as well as total mesophilic aerobic bacteria. Similarly, no significant effect on sensory properties. Whereas, the acidity, pH, and fat were significantly changed by LM treatment	(30)
6	An edible film of CH and LM on Mongolian cheese surface by electrostatic shelf-assembly technology was used.	The coating showed a broad-spectrum bacteriostatic effect. Significant enhancement in shelf life, delayed fat precipitation, and quality of cheese.	(179)
7	Edible films based on FG + sodium alginate (SA) with varied concentrations of carvacrol on Chinese sea bass filets during cold storage	The films containing carvacrol at concentrations of 1.0 or 2.0 mg/mL remarkably decreased the degree of microbial deterioration, total volatile basic nitrogen (TVB-N) content, and adenosine triphosphate (ATP) decomposition (*K* value), as well as maintained the quality (e.g., freshness) of sea bass fillets	(27)
8	Linseed mucilage + pectin-based film impregnated with calcium chloride and titanium dioxide	Surface films have no cracks and agglomerates as observed from SEM. Reduction in swelling index due to cross-linking of calcium chloride. Increased duration of biodegradation of films with an increase in titanium dioxide	(187)
9	LM + PVA + chitosan films fabricated with gamma radiation	LM + PVA/cs blend improves mechanical and thermal stability Gamma radiation improved the performance of film	(162)
10	LM + cardamom and coapaiba	Modification in the color of film is observed but thickness remains constant With the incorporation of cardamom and copaiba, there is an increase in solubilization time from 9 to 12 min Increase in antioxidant activity DPPH, ABTS Decrease in hardness (39–70%), deformation (49–78%), and fracture ability (39–82%)	(188)
11	LM + sodium alginate film prepared and added with norbixin and W03 nanoparticles	Antioxidant-rich film was formed. Controlled and adequate release of norbixin was found in film	(182)
12	Simple one pot non-toxic method to develop packaging material	The LM conjugate has a high water-holding capacity of 87–62%. The LM shows Young’s modulus of 1–3 GPa, glass transition temperature between 49 and 103°C	(183)
13	LM + *piper betel* extract edible films	Film exhibited good barrier activity, antioxidant, and antimicrobial activity with a higher diameter of inhibition zone compared to the control	(14)

## 8 Conclusion and future perspectives

Linseed mucilage (LM) studies have unveiled an opportunity to explore it as an abundant source of dietary fibers and bioactive compounds. The application of LM as a functional food is supported by its remarkable health-promoting properties. The conventional extraction methods are harsh, leading to deterioration of the quality of LM and thus a reduction in its health advantages. This can be overcome by non-conventional pretreatment approaches prior to extraction that aim to improve the mucilage quality, thus promoting their industrial utilization. The addition of LM to food products has been made possible owing to its excellent functional properties, namely water-holding, gelling, thickening, binding, texturing, foaming, emulsion formation, and stabilizing characteristics. The protective properties exhibited by LM aid in increasing the shelf life of food products, thus suggesting its potential application as a food packaging film and coating while maintaining its sensory properties. The use of LM opens opportunities for greener, innovative prospects and emerging developments in the field of food packaging by providing a potential solution for plastic packaging waste. Subsequent research efforts could be directed toward enhancing the mechanical strength of LM films and improving their microbial resistance to provide a complete solution as biodegradable packaging on a commercial scale.

## Author contributions

MC: Writing – original draft, Writing – review & editing, Conceptualization, Data curation, Formal analysis, Investigation, Resources, Visualization. RC: Writing – original draft, Writing – review & editing, Conceptualization, Data curation, Formal analysis, Investigation, Project administration, Supervision, Validation. BT: Writing – original draft, Writing – review & editing. SH: Writing – original draft, Writing – review & editing. PS: Writing – original draft, Writing – review & editing. AD: Writing – review & editing. AP: Writing – review & editing, Software.
